# Dietary Apigenin Exerts Immune-Regulatory Activity *in Vivo* by Reducing NF-κB Activity, Halting Leukocyte Infiltration and Restoring Normal Metabolic Function

**DOI:** 10.3390/ijms17030323

**Published:** 2016-03-01

**Authors:** Horacio Cardenas, Daniel Arango, Courtney Nicholas, Silvia Duarte, Gerard J. Nuovo, Wei He, Oliver H. Voss, M. Elba Gonzalez-Mejia, Denis C. Guttridge, Erich Grotewold, Andrea I. Doseff

**Affiliations:** 1 Department of Physiology and Cell Biology, the Heart and Lung Research Institute, the Ohio State University, Columbus, OH 43210, USA; horacio.cardenas@northwestern.edu (H.C.); dany33co@gmail.com (D.A.); nicholas.40@buckeyemail.osu.edu (C.N.); duartesanmiguel.1@osu.edu (S.D.); elba.gonzalezmejia@gmail.com (M.E.G.-M.); 2 Department of Molecular Genetics, the Ohio State University, Columbus, OH 43210, USA; ollivoss39@gmail.com (O.H.V.); Grotewold.1@osu.edu (E.G.); 3 Molecular Cellular and Developmental Biology Graduate Program, the Ohio State University, Columbus, OH 43210, USA; he.115@osu.edu; 4 Nutrition Graduate Program, the Ohio State University, Columbus, OH 43210, USA; 5 Comprehensive Cancer Center, the Ohio State University, Columbus, OH 43210, USA; gerard.nuovo@osumc.edu (G.J.N.); denis.guttridge@osumc.edu (D.C.G.); 6 Center for Applied Plant Sciences, the Ohio State University, Columbus, OH 43210, USA

**Keywords:** apoptosis, flavonoids, apigenin, leukocytes, NF-κB, inflammation, sepsis, mitochondria, cardiac dysfunction

## Abstract

The increasing prevalence of inflammatory diseases and the adverse effects associated with the long-term use of current anti-inflammatory therapies prompt the identification of alternative approaches to reestablish immune balance. Apigenin, an abundant dietary flavonoid, is emerging as a potential regulator of inflammation. Here, we show that apigenin has immune-regulatory activity *in vivo*. Apigenin conferred survival to mice treated with a lethal dose of Lipopolysaccharide (LPS) restoring normal cardiac function and heart mitochondrial Complex I activity. Despite the adverse effects associated with high levels of splenocyte apoptosis in septic models, apigenin had no effect on reducing cell death. However, we found that apigenin decreased LPS-induced apoptosis in lungs, infiltration of inflammatory cells and chemotactic factors’ accumulation, re-establishing normal lung architecture. Using NF-κB luciferase transgenic mice, we found that apigenin effectively modulated NF-κB activity in the lungs, suggesting the ability of dietary compounds to exert immune-regulatory activity in an organ-specific manner. Collectively, these findings provide novel insights into the underlying immune-regulatory mechanisms of dietary nutraceuticals *in vivo*.

## 1. Introduction

Inflammation is a normal central mechanism of host immune surveillance in response to infections. However, dysregulated inflammation leads to systemic activation of the host immune response, tissue injury and, ultimately, organ failure [1]. Hence, dysregulated inflammation has been implicated in the pathophysiology of several diseases, including acute lung injury and sepsis [2]. High morbidity and mortality are associated with these diseases, and current successful therapies are limited [3,4,5,6]. Thus, there is a great need to identify new therapeutic approaches.

Lipopolysaccharide (LPS) is a component of Gram-negative bacteria cell wall that promotes inflammation and the production of inflammatory mediators. Increased blood levels of endotoxin have been reported in acute lung injury and sepsis patients [7]. Dysregulated inflammation induces metabolic changes, characterized by cardiac dysfunction, the alteration of vascular functions, the production of inflammatory mediators and excessive apoptosis [8,9,10]. These contribute to the development of lung injury and impaired lung and heart function [11]. LPS triggers a complex signal transduction cascade leading to the activation of the transcription factor NF-κB [12], a central regulator of inflammation [12]. NF-κB promotes the expression of cytokines and chemokines [13,14]. Among them, KC (keratinocyte chemoattractant) and MIP-2 (macrophage inflammatory protein-2) are potent LPS-induced neutrophil chemoattractant factors, highly expressed in lung injury [15,16,17]. In addition, metabolic alterations are central to acute inflammation, and causative links have been reported between sepsis-induced mortality and extensive mitochondrial dysfunction in ill patients [6,18]. Moreover, excessive apoptosis has been described in sepsis and endotoxemia patients [19,20,21]. Thus, approaches that reduce cell death and restore normal inflammatory responsiveness and metabolic function have been proposed as promising treatments for sepsis [22,23,24,25].

Flavonoids, plant phenolic compounds constituting the largest class of non-essential nutrients in our diet, have been shown to have anti-cancer and anti-inflammatory activities [26,27,28]. The flavonoid apigenin is found at high levels in parsley and celery, both abundant in the Mediterranean diet. Apigenin regulates inflammatory mediators, including IL-1β and TNF-α, in both human and mouse cell lines [29,30]. We found that apigenin reduced pro-inflammatory cytokine production by inhibiting NF-κB phosphorylation in macrophages and reduced neutrophil chemotaxis *in vitro* [31,32]. Moreover, apigenin reduced the apoptosis of endothelial cells cultured with LPS by restoring normal mitochondrial Complex I activity [33]. Celery-based diets formulated to increase apigenin absorption effectively reduced LPS-induced NF-κB activity, decreasing TNF-α in macrophage cell lines [34] and inflammatory microRNAs *in vivo* [35]. Despite these advances, the mechanisms underlying the immune-regulatory activity of apigenin *in vivo* remained poorly understood.

In this study, we investigated the immune-regulatory mechanisms of apigenin *in vivo*. We found that apigenin protected mice against LPS-induced mortality even during a 30-day period, suggesting a long-lasting effect in restoring homeostasis. Interestingly, apigenin-induced survival did not correlate with reduced splenocyte apoptosis, as indicated by histological, TUNEL assays, immune-histochemistry of active caspase-3 and flow cytometry studies. However, we found that apigenin restored normal cardiac function and metabolic mitochondrial Complex I activity in LPS-treated mice. Using NF-κB-luciferase transgenic mice, we showed that apigenin effectively reduced LPS-induced NF-κB activity *in vivo* in an organ-specific manner. Apigenin also reduced LPS-induced neutrophil lung infiltration and chemokine production. These effects were accompanied by a decrease of LPS-induced lung apoptosis and the restoration of normal lung architecture. Together, these studies provide evidence of the mechanisms underlying the immune-regulatory activity of apigenin *in vivo* and providing new insights in the ability of dietary compounds to confer organ-specific regulation of NF-κB. Our findings suggest that dietary interventions have novel implications for the regulation of inflammatory diseases.

## 2. Results

### 2.1. Apigenin Induces Long-Term Survival in Lipopolysaccharide (LPS)-Treated Mice

**Figure 1:**
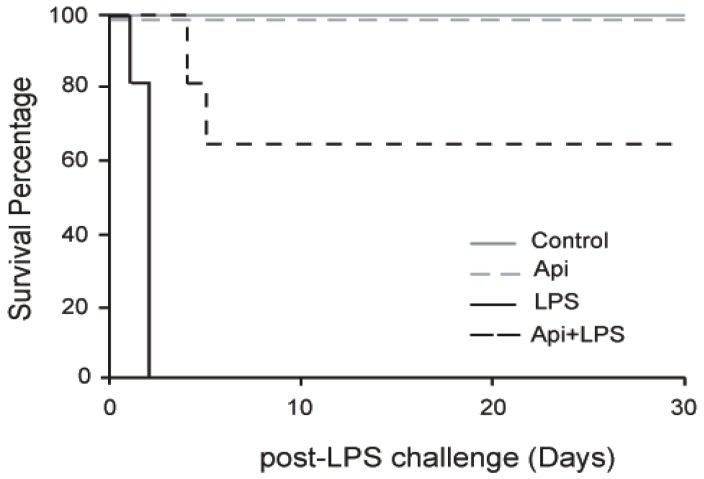
Apigenin confers long-term survival to lipopolysaccharide (LPS)-treated mice. Kaplan–Maier survival curves were generated for mice receiving vehicle (Phosphate Buffered Saline (PBS) + Dimethyl Sulfoxide (DMSO); control), 50 mg/kg apigenin alone (Api), apigenin vehicle 3 h before injection of 37.5 mg/kg LPS (LPS) and apigenin 3 h before LPS injection (Api + LPS). Mice were monitored every 4 h for the first 72 h and daily for 30 days. *N* = 8 mice for each condition; * *p* < 0.05, LPS compared to Api + LPS.

To evaluate the effect of apigenin on long-term protection from inflammation, mice were injected with apigenin (50 mg/kg) or vehicle control 3 h prior to LPS (37.5 mg/kg). No mortality occurred in mice receiving apigenin (Api mice) or vehicle only (control mice; Figure 1). All mice pretreated with diluent DMSO and receiving LPS (LPS mice) died after 30 h, in agreement with previous reports [32]. However, 70% of LPS-treated mice that also received apigenin (Api + LPS mice) survived throughout the 30-day evaluation period. These results indicate that administration of apigenin supports long-lasting survival upon acute inflammation.

### 2.2. Apigenin Prevents LPS-Induced Cardiac Dysfunction and Restores Normal Mitochondrial Complex I Activity

**Figure 2:**
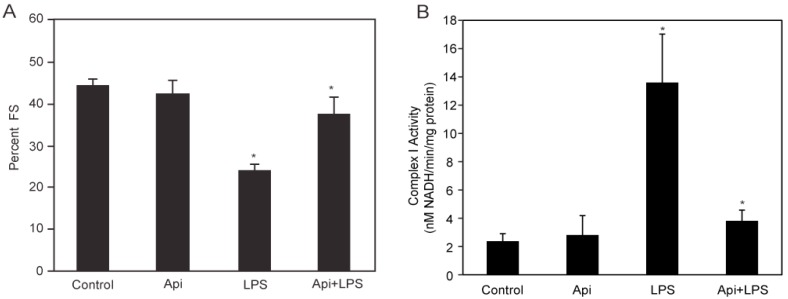
Apigenin restores normal cardiac and mitochondrial Complex I function. (**A**) The percent fractional shortening (%FS) in control, Api, LPS and Api + LPS mice was determined by M-mode echocardiography at 24 h. Data represent the mean ± SEM of four independent experiments using *N* = 5 mice for each condition (20 mice per experimental condition; * *p* < 0.02 *vs.* LPS); (**B**) Mitochondrial Complex I activity was assessed in heart lysates from all four groups. Changes in Nicotinamide Adenine Dinucleotide (NADH) absorbance at 340 nm were determined every 10 s for 6 min. Data represent the mean ± SEM, *N* = 5 mice; * *p* < 0.02 *vs.* LPS).

LPS-induced inflammation is characterized by cardiac dysfunction [10]. To determine whether apigenin affected cardiac function in LPS mice, serial echocardiography (ECG) was performed. Fractional shortening (FS) was decreased by ~50% in LPS mice, compared to control or Api animals at 24 h (Figure 2A). Apigenin administration increased the percentage of fractional shortening in LPS-treated mice by two-fold, reaching levels found in control and Api mice (Figure 2A).

To understand the mechanisms underlying the protective effects of apigenin on cardiac function, we examined the effect of apigenin on mitochondrial Complex I activity during LPS stimulation. Mitochondrial lysates from hearts of LPS mice showed a ~5-fold increase in Complex I activity, as compared to controls (Figure 2B). Mitochondrial Complex I activity was reduced to control levels in Api + LPS mice (Figure 2B). Apigenin alone had no effect on mitochondrial Complex I activity. These results showed that apigenin prevents cardiac dysfunction and restores normal mitochondrial metabolic function, suggesting the ability of apigenin to modulate cardiac homeostasis during inflammation.

### 2.3. Apigenin-Induced Survival in LPS-Treated Mice Is Independent of Splenocyte Apoptosis

**Figure 3:**
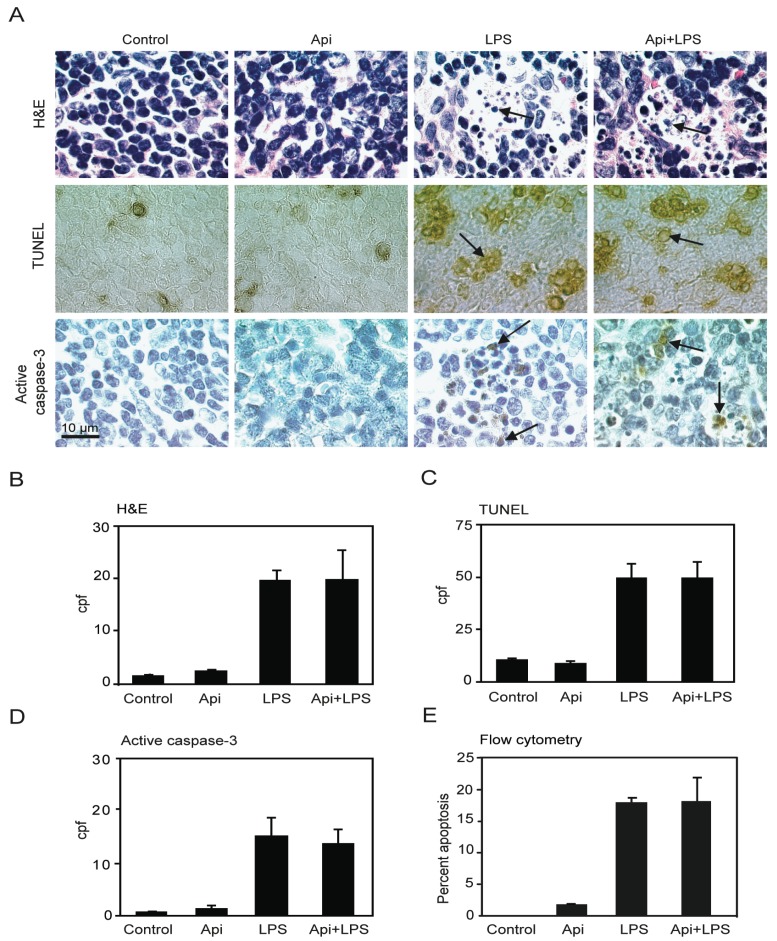
Apigenin has no effect on LPS-induced splenocyte apoptosis. (**A**) H&E staining (**top**), TUNEL (**middle**) and immunohistochemistry (IHC) for active caspase-3 (**bottom**) in spleens from control, Api, LPS and Api + LPS mice at 24 h. In all pictures scale bars represent 10 μm, arrows indicate apoptotic cells; (**B**) Quantification of apoptotic cells as determined by H&E; (**C**) Number of apoptotic cells detected by TUNEL; (**D**) Number of apoptotic cells detected by staining with anti-active caspase-3 specific antibodies; (**E**) Percentage of apoptotic cells determined by Annexin V/Propidium Iodide (PI) staining using flow cytometry. Data represent the mean ± SEM (*N* = 5 mice for each condition; no statistical difference between LPS and Api + LPS).

The increase of the number of cells undergoing apoptosis in the spleen has been suggested as a key factor of immune-dysregulation during acute inflammation [20]. Previous studies using different animal models showed that LPS-induced inflammation leads to an increase in splenocyte apoptosis [36,37]. To examine whether the survival conferred by apigenin in LPS-mice was due to a reduction of apoptosis, four independent approaches were used to evaluate cell death in spleens. A ~10-fold increase in apoptotic cells was observed in spleens of LPS mice as compared to controls or Api mice at 24 h (Figure 3A,B; H&E, LPS *vs.* control). The number of apoptotic cells in Api + LPS mice was high and comparable to the levels of apoptosis observed in LPS mice (Figure 3A,B; H&E, Api + LPS *vs.* control). Low basal levels of apoptosis were observed in spleens from both control and Api mice (Figure 3A,B; H&E, control and Api).

TUNEL assays showed a ~4-fold increase of apoptosis in spleens in LPS mice, as compared to the control or Api groups. Api + LPS had similar levels of apoptotic cells compared to LPS mice (Figure 3A,C). Next, apoptosis was evaluated by immunohistochemistry using an antibody that recognizes active caspase-3, a well-accepted marker of apoptosis. A ~10-fold increase in cells stained positive for active caspase-3 was found in both LPS and Api + LPS mice, as compared to the control and Api groups (Figure 3A,D). Finally, apoptosis was quantified by Annexin V/Propidium Iodide (PI) staining. Approximately 20% of splenocytes were apoptotic at 24 h in both LPS and Api + LPS mice (Figure 3E). This represents an ~20-fold increase relative to control or Api mice (Figure 3E). Collectively, these findings demonstrate that the survival conferred by apigenin during inflammation is not mediated by a reduction of apoptosis in the spleens.

### 2.4. Apigenin Regulates LPS-Induced NF-κB Activity in Vivo in Lungs

**Figure 4:**
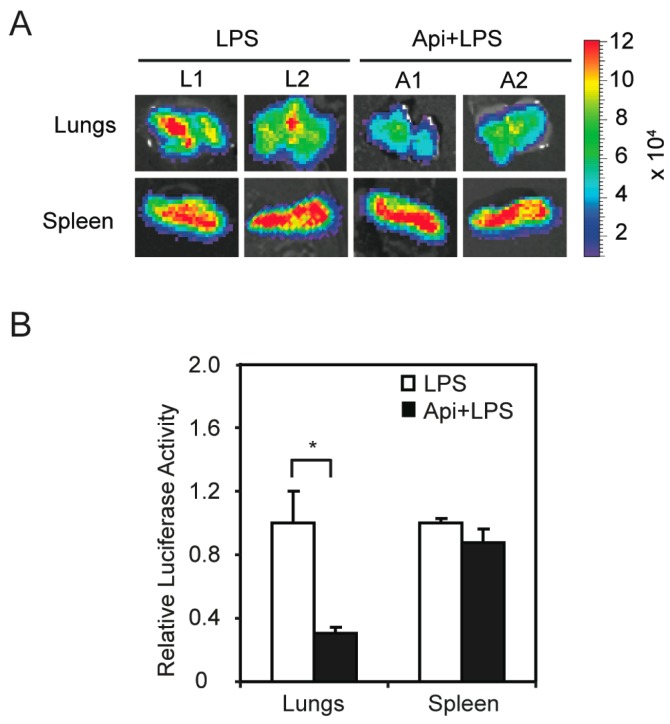
Apigenin inhibition of LPS-induced NF-κB activity *in vivo* shows organ specificity. NF-κB-Responsive Elements-luciferase transgenic mice were treated with apigenin (50 mg/kg of body weight) or vehicle 3 h prior to the administration of 37.5 mg/kg LPS for 6 h. (**A**) Lungs and spleens were excised, imaged and luciferase activity expressed as photons/s/cm^2^/steradian; (**B**) Luciferase activity was assayed in tissue homogenates and expressed as luciferase activity relative to LPS. Values represent the means ± SEM (*N* = 5; * *p* < 0.05).

The finding that apigenin improved acute inflammation without affecting apoptosis in spleens prompted us to investigate its mechanisms of action. We previously showed that apigenin reduces LPS-induced phosphorylation of the NF-κB p65 subunit in macrophages [32]. To examine if apigenin modulates NF-κB activity *in vivo*, we used transgenic mice expressing luciferase under the control of NF-κB responsive elements. In agreement with previous reports [38], transgenic mice treated with LPS showed a significant increase of luciferase expression, an indicator of NF-κB activity, at 6 h, as compared to control mice (data not shown). Apigenin decreased LPS-induced luciferase expression in lungs, but had no effect on splenic luciferase levels (Figure 4A). Consistent with these findings, luciferase activity assays in tissue homogenates showed that apigenin decreased the expression of luciferase in lungs of LPS mice by three-fold, but had no effect on NF-κB activity in the spleens (Figure 4B). These findings provide evidence that apigenin modulates NF-κB activity in an organ-specific manner *in vivo*.

### 2.5. Apigenin Decreases LPS-Induced Neutrophil Accumulation in the Lungs

**Figure 5:**
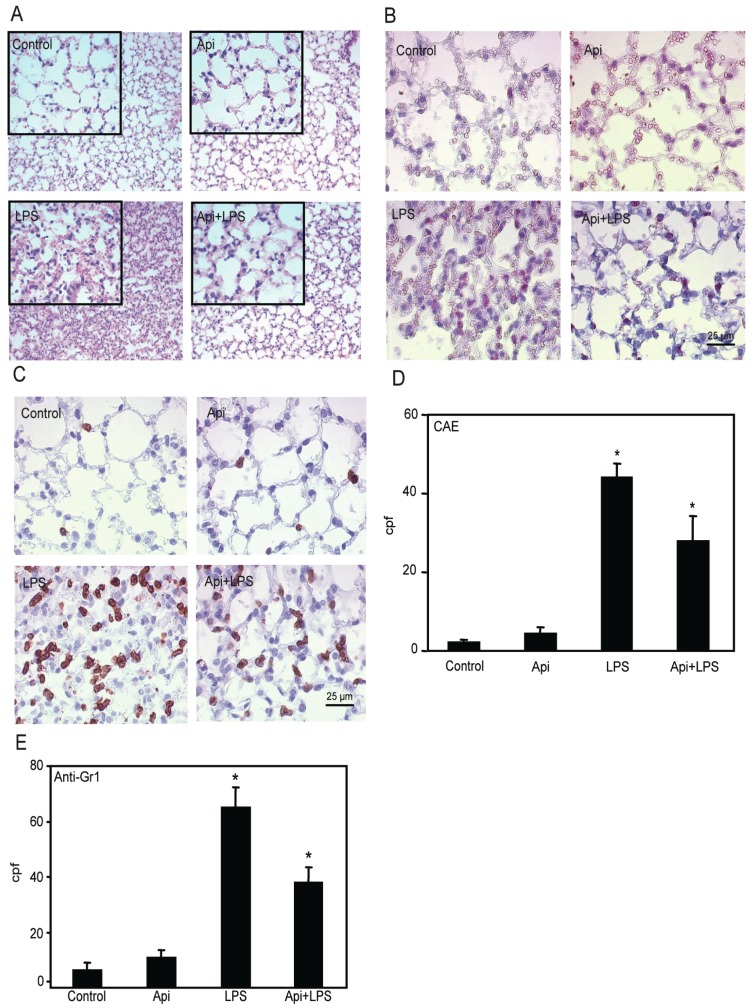
Apigenin decreases LPS-induced neutrophil infiltration in the lungs. Lungs from mice receiving Api + LPS, LPS, Api or vehicle (control) were collected at 24 h post-challenge. (**A**) H&E staining; (**B**) Chloroacetate esterase (CAE) staining; (**C**) Lungs stained with anti-7/4 antibodies; (**D**) Leukocyte infiltration determined by counting CAE-positive cells per high power field (cpf) in lungs used in (**B**); (**E**) Number of infiltrated neutrophils as determined by counting stained cells per cpf in in lungs used in (**C**). Data represent the mean ± SEM (*N* = 9 mice for each biological condition; * *p* < 0.05 *vs.* LPS). Scale bars for all figures are 25 μm and 100 μm for insets.

Based on the effectiveness of apigenin in modulating NF-κB activity in the lungs, we next examined its ability to regulate inflammation in this organ. In agreement with previous studies [39], we found increased neutrophil infiltration in lungs of LPS mice as compared to control or Api mice (Figure 5A). Apigenin significantly attenuated this effect.

Lung architecture, abnormal in LPS mice, was restored to normal in Api + LPS animals. To further evaluate the effect of apigenin on leukocyte infiltration, lungs were stained with chloroacetate esterase (CAE). LPS mice showed an ~8-fold increase in neutrophil infiltration as compared to control or Api groups (Figure 5B,D). Neutrophil infiltration was significantly decreased in Api + LPS mice as compared to LPS animals (Figure 5B,D). Moreover, lung tissue sections stained with anti-7/4-antibodies, a neutrophil-specific marker, showed that apigenin significantly decreased neutrophil infiltration in LPS-treated mice (Figure 5C,E).

### 2.6. Apigenin Decreases LPS-Induced Lung Neutrophil Chemoattractant Macrophage Inflammatory Protein-2 (MIP-2)

**Figure 6:**
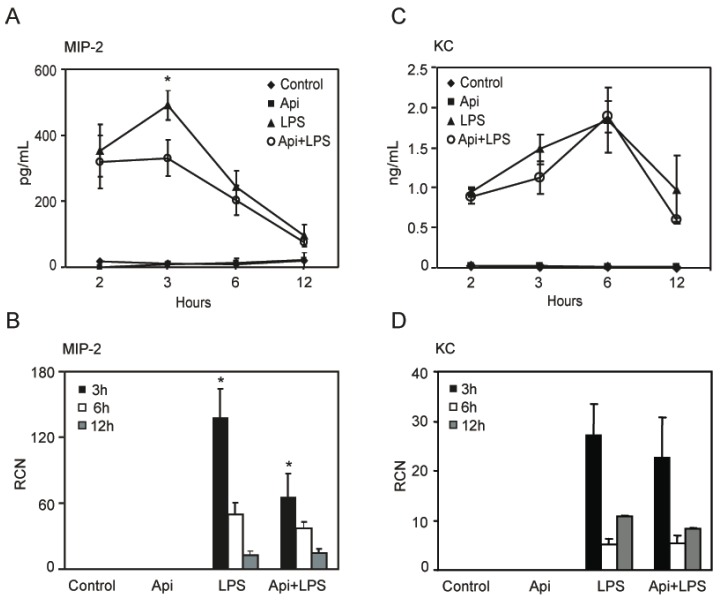
Apigenin reduces LPS-induced lung inflammatory chemokines. Bronchoalveolar lavage fluid (BALF) and whole lungs were obtained from control, Api, LPS and Api + LPS mice. (**A**,**C**) Macrophage inflammatory protein-2 (MIP-2) and keratinocyte chemoattractant (KC) protein expression was determined by ELISA in BALF; (**B**,**D**) MIP-2 and KC steady-state mRNA levels in whole lung were determined by quantitative RT-PCR. Values represent the means ± SEM (*N* = 6 mice for each biological condition and time point; * *p* < 0.05, compared to LPS-treated mice at the same time point). RCN, relative copy number.

To define the mechanisms by which apigenin reduced lung neutrophil infiltration, we assessed the level of KC and MIP-2, two neutrophil chemoattractant factors. LPS increased MIP-2 levels in bronchoalveolar lavage fluid (BALF) at 2 h, reaching a maximum at 3 h and decreasing to basal levels, found in controls or mice treated with apigenin alone, by ~12 h (Figure 6A). Apigenin treatment resulted in a significant reduction of LPS-induced MIP-2 at 3 h, at the maximum peak reached during inflammation (Figure 6A). Consistent with these findings, apigenin decreased LPS-induced expression of MIP-2 mRNA in lung tissues (Figure 6B). In addition, an increase of KC was observed in BALF by ELISA and in lung tissues by quantitative RT-PCR, as compared to control or apigenin mice (Figure 6C,D). Apigenin had no effect on LPS-induced expression of KC mRNA or protein (Figure 6C,D). These findings suggest that apigenin ameliorates lung inflammation by decreasing neutrophil infiltration through the modulation of specific chemoattractant factors.

### 2.7. Apigenin Reduced LPS-Induced Lung Apoptosis

**Figure 7:**
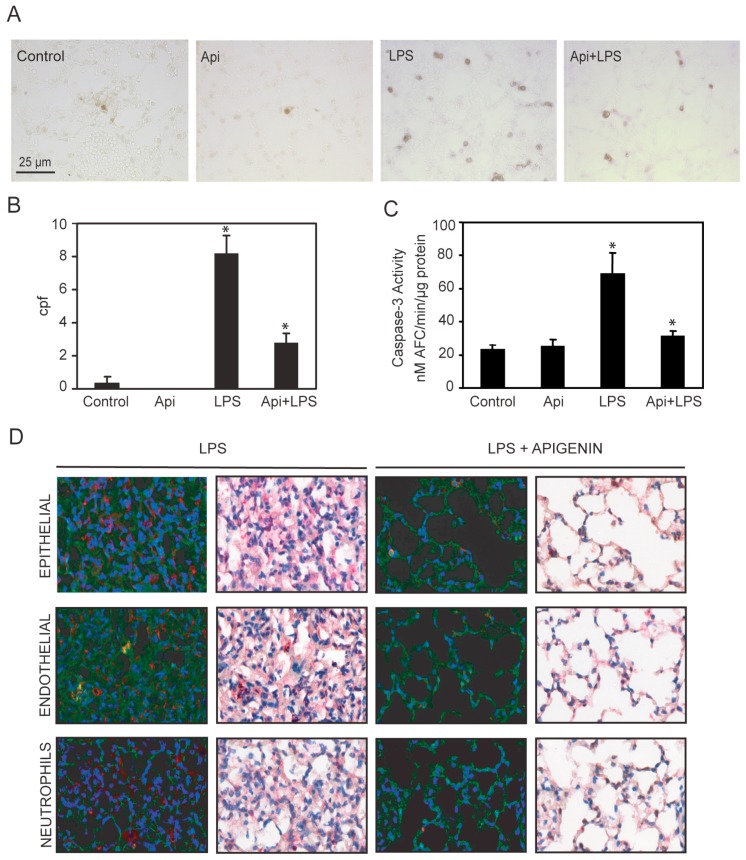
Apigenin reduces LPS-induced apoptosis in the lungs. Lungs from mice treated with LPS, control, apigenin (Api) or apigenin 3 h prior to LPS (Api + LPS) were obtained at 24 h. (**A**) The number of apoptotic cells was determined by the TUNEL assay and expressed as the mean ± SEM of the numbers of stained cells per 1000× microscopic field (cpf) (*N* = 4 mice for each biological condition; * *p* < 0.01 *vs.* LPS), scale bars: 25 μm for all figures; (**B**,**C**) Caspase-3 activity was determined by the DEVD-AFC assay using lung lysates as described in materials and methods. Values represent the means ± SEM (*N* = 4 mice for each biological condition; * *p* < 0.05 *vs.* LPS); (**D**) Lung tissue immuno-histochemistry using antibodies specific for active caspase-3 in combination with anti-CD31, anti-cytokeratin or anti-7/4 antibodies specific for epithelial, endothelial and neutrophils, respectively. Scale bars, 100 μm.

Dysregulated inflammation induces lung damage characterized by an increase in apoptosis [40]. To investigate the effect of apigenin in inflammation-induced apoptosis, we evaluated the number of apoptotic cells in the lungs. An ~8-fold increase in apoptotic cells was observed in LPS mice as compared to controls or Api animals (Figure 7A,B). Administration of apigenin reduced by ~3-fold the number of apoptotic cells in LPS-treated mice (Figure 7A,B). Api mice showed only a basal low number of apoptotic bodies, similar to levels found in control mice (Figure 7A,B). In addition, we evaluated the level of caspase-3 activity in lung tissue lysates. LPS increased caspase-3 activity ~4-fold as compared to control or Api mice (Figure 7C). Apigenin reduced caspase-3 activity in LPS-treated mice to levels found in control mice (Figure 7C).

To gain insight into the cells protected by apigenin, lungs were co-stained with antibodies to active caspase-3 and cell-type-specific antigens. Neutrophils and epithelial cells stained with anti-7/4 and anti-cytokeratin antibodies, respectively, showed no co-staining with active caspase-3 (Figure 7D, see yellow) in lungs of LPS mice.

In contrast, in LPS mice, endothelial cells stained with anti-CD31 antibodies showed co-staining with active caspase-3 (Figure 7D, see yellow). Api mice showed normal lung architecture restored and no staining with active caspase-3 antibodies, indicating a reduction of endothelial cell apoptosis (Figure 7D). These findings suggest that apigenin decreases lung endothelial cell apoptosis and restores normal lung architecture.

## 3. Discussion

Long-term use of current anti-inflammatory drugs is associated with adverse effects and high medical costs. Thus, there is increasing interest in identifying alternative immune-regulators. Dietary compounds might offer beneficial health effects, at low cost and with minimal or in the absence of adverse effects. The present study shows the efficacy of the dietary compound apigenin to ameliorate inflammation and restoring immune-balance *in vivo*.

We have previously shown that apigenin improves the survival of mice during the first week after LPS administration [32]. The present study shows that apigenin reduced mortality associated with inflammation by ~70% even after 30 days, suggesting that the administration of apigenin induces recovery from the lethal effects of endotoxemia (Figure 1). Metabolic and mitochondrial dysfunction during endotoxin shock are involved in tissue damage [41]. Mitochondrial Complex I is considered a major site of electron leaking, leading to the formation of superoxide and hydrogen peroxide [42], key contributors to tissue damage. We found that apigenin restored normal cardiac function in LPS-treated mice (Figure 2A). Cellular studies showed that flavonoids modulate mitochondrial respiratory Complex I [43]. Recently, we reported that in endothelial cell cultures, apigenin restores LPS-induced dysregulated Complex I activity, thereby preventing mitochondrial damage and reducing excess free radical production [33]. Here, we show that apigenin restored basal mitochondrial Complex I activity in hearts of LPS-treated mice (Figure 2B), suggesting its relevance in modulating metabolic balance.

Inflammation induced by LPS or cecal ligation and puncture (CLP) leads to increased apoptosis in lymphoid organs, including the spleen [36,37,44]. Excessive apoptosis has been described in sepsis and endotoxemia patients [19,20,21]. Consistent with this, we observed increased apoptosis in the spleens of LPS-treated mice. Several studies showed that decreasing cell death, either by overexpressing anti-apoptotic genes or administering caspase or protease inhibitors [24,45], improves survival. Intriguingly, however, apigenin did not alter cell death in spleens (Figure 3), despite its high efficacy in reducing mortality. These results provide evidence that apigenin, despite lacking the ability to affect spleens, significantly improves survival, suggesting other critical mechanisms that may contribute to favorable outcomes in sepsis.

Dysregulated inflammation leads to increased NF-κB activity and inflammatory chemokines and chemoattractant levels. Thus, inhibition of the LPS-stimulated signal transduction cascade has been proposed as a promising target for the treatment of sepsis [22,23]. We previously showed that apigenin reduced NF-κB activity in macrophages [32]. Here, we demonstrate that apigenin reduces LPS-induced NF-κB activity *in vivo*, as supported by the reduced level of luciferase activity in transgenic mice that express luciferase under the control of NF-κB responsive elements (Figure 4). Interestingly, these effects seem to be organ specific, as apigenin reduced NF-κB activity in lungs, but had no effect in spleens (Figure 4) or hearts (data not shown). These results may reflect the ability of apigenin to influence cardiac function through a non-canonical NF-κB pathway, which is independent of the p65 subunit and, therefore, would not be recognized using this transgenic model. Alternatively, it may be attributed to NF-κB regulation acting, however, in a paracrine manner or to an NF-κB-independent mechanism. So far, NF-κB and its role in metabolism remained poorly understood. Recent studies suggested that NF-κB organizes energy metabolism networks by balancing glycolysis and mitochondrial respiration [46], yet the mechanisms underlying NF-κB with mitochondrial Complex I are yet to be determined and were outside the scope of this work. Recently, we identified isocitrate dehydrogenase (IDH), a major regulatory point in the tricarboxylic acid (TCA) cycle, as a direct target of apigenin [47]. Thus, an additional alternative is that apigenin, by altering IDH activity, counteracts mitochondrial dysregulation in hearts, whereas the inflammatory “cytokine storm” is regulated by altering NF-κB in lungs. Future studies in this area will be needed.

Recent studies showed that apigenin reduced acute lung injury in a model of intratracheal LPS administration [48]. Excessive neutrophil infiltration during inflammation has been implicated in lung injury [49]. Increased neutrophil infiltration was previously reported in lungs of LPS-treated mice [15,16]. In agreement with these observations, a significant increase of neutrophils was found in lungs of mice treated with LPS. Importantly, we found that apigenin reduced neutrophil infiltration (Figure 5). KC and MIP-2 are potent neutrophil chemoattractants, sharing a common receptor, CXCR2 [50,51]. Previous studies showed that LPS increased MIP-2 and KC mRNA levels in a time-related manner with a maximum of ~3 to 6 h [52]. In agreement with these studies, we found a significant transient increase of both MIP-2 and KC occurring at ~3 h after LPS and reaching basal levels at later times (Figure 6). However, no changes were observed in KC. Inhibition of neutrophil infiltration and a decrease in MIP-2 levels have been reported in LPS-treated mice receiving fisetin and tricetin [53], suggesting the efficacy of other flavones in modulating inflammatory mediators. Differences in MIP-2 and KC have been previously reported, including their differential kinetics and response to glucocorticoids [54]. In addition, MIP-2 has a 72-fold greater affinity for CXCR2 than KC [55], suggesting a key role of MIP-2 in altering neutrophil infiltration. Accordingly, inhibition of MIP-2 in the lungs using MIP-2 antiserum decreased neutrophils’ influx by 60% in a model of bacterial-induced inflammation [56]. KC and MIP-2 seem to be expressed preferentially by different cell types. *In situ* studies showed MIP-2 expression mainly in neutrophil and macrophages, whereas KC was preferentially expressed in endothelial cells [57]. In addition, cytokine array studies found high expression of KC in lung epithelial cells during LPS stimulation, accompanied by a low increase of MIP-2 [58]. Moreover, in models of lung injury, MIP-2 levels were found to be ~20-times higher in macrophages and negligible in epithelial cells, whereas KC was exclusively found in epithelial cells [59]. Thus, the effectiveness of apigenin reducing MIP-2, but not KC may reflect its ability to affect inflammation in a more selective cell-specific manner.

Our studies showed that apigenin restores normal lung architecture in LPS-treated mice (Figure 7). In zebra fish, apigenin was found to increase neutrophil apoptosis contributing to resolve inflammation [60]. We found a significant reduction of neutrophil infiltration accompanied by decreased MIP-2 expression rather than an increase in neutrophil apoptosis. However, apigenin reduced apoptosis in the lungs, an effect likely due to its protection of endothelial cells (Figure 3). Despite its ability to regulate apoptosis in lungs, apigenin had no effect in reducing apoptosis in spleens (Figure 3 and Figure 7).

In summary, we showed that apigenin decreases LPS-induced NF-κB activity at specific organs, which may contribute to restore the homeostatic balance during inflammation, reducing lung neutrophil infiltration and apoptosis. Collectively, these findings provide novel insights into the underlying immune-regulatory mechanisms of dietary compounds *in vivo*.

## 4. Experimental Section

### 4.1. Reagents

Apigenin (4′,5,7-trihydroxyflavone), bacterial lipopolysaccharide (LPS, serotype 0127:B8, 1 × 10^6^ EU/mg) and diluent DMSO were from Sigma (St. Louis, MO, USA). Luciferin was purchased from Caliper Life Sciences (Hopkinton, MA, USA). The luciferase Assay Kit was from Promega (Fitchburg, WI, USA). Apigenin was dissolved in DMSO and LPS in PBS (Phosphate Buffered Saline). Control mice refer to animals receiving PBS and DMSO diluents. LPS mice received LPS and diluent DMSO.

### 4.2. Mice Experiments, Imaging and Luciferase Activity

Male C57BL/6J mice were purchased from Harlan Teklad (Madison, WI, USA) and BALB/C-Tg (NF-κB-RE-luc)-Xen mice, which systemically express luciferase under the control of NF-κB, were obtained from Taconic (Hudson, NY, USA). NF-κB-driven luciferase expression in different mice organs has been previously reported by our group and others [38]. All male mice were used at 6 to 8 weeks of age after a 10-day acclimatization, as previously described [32]. All procedures were approved by The Ohio State University Institutional Animal Care and Use Committee (IACUC-OSU) project 2007A0208-R2 (14 November 2015). Mice were injected intraperitoneally (i.p.) with apigenin (50 mg/kg of body weight), or diluents DMSO (Dimethyl Sulfoxide)/PBS 3 h prior to administration of LPS (37.5 mg/kg), or PBS for the times indicated in the Results Section. For detection of luciferase, mice were administered D-luciferin (150 mg/kg body weight) by i.p. anesthetized using 1% isoflurane (IsoFlo, Abbott Labs, Chicago, IL, USA) and immediately euthanized. Organs were excised and imaged using an ultrasensitive camera consisting of an image intensifier coupled to a charge-coupled device (CCD; Xenogen IVIS Imaging System; Xenogen, Alameda, CA, USA). The images were processed using the Living Image Software (Caliper Life Sciences, Hopkinton, MA, USA) and expressed as photons/s. For luciferase enzymatic activity, ~50 mg of tissue were homogenized in 400 µL of luciferase lysis buffer using a Brinkmann Polytron PTA 7K1 homogenizer (Brinkmann instruments Inc., Westbury, NY, USA). Luciferase activity was assessed in homogenates using the Luciferase Assay Kit (Promega). Data were normalized to protein concentration and represented as luciferase activity relative to LPS.

### 4.3. Lungs and Bronchoalveolar Lavage

Bronchoalveolar lavage fluid (BALF) was obtained as previously described [61]. Briefly, mice were sacrificed and immediately underwent tracheotomy. The trachea was cannulated and lavage performed using 0.5 mL PBS three times. The lungs were inflated with PBS (~20 cm pressure); the left lung was immediately ligated, excised, fixed in 10% formalin and embedded in paraffin for histological evaluation. The remaining lobes were snap frozen in liquid nitrogen and stored at −80 °C for RNA isolation. BALF was centrifuged to remove cells and stored at −80 °C.

### 4.4. Assessment of Apoptosis in Tissue

Spleens and lungs were harvested at 24 h, and apoptosis was quantified by counting apoptotic bodies in sections stained with hematoxylin and eosin (H&E) and by the TUNEL assay using the ApopTag Plus apoptosis detection kit (#S7101, Chemicon International-Millipore, Billerica, MA, USA) following the manufacturer’s instructions. In addition, immunohistochemistry (IHC) was performed to detect active caspase-3 in spleens. Briefly, sections were de-paraffinized and rehydrated, incubated for 20 min with antigen retrieval solution (#H3300, Vector Labs, Burlingame, CA, USA) and blocked for 1 h at room temperature (RT) with PBS containing 5% goat serum (GIBCO/Invitrogen, Carlsbad, CA, USA). Sections were then incubated for 2 h at RT with anti-active-caspase-3 antibodies (Clone #9661, Santa Cruz Biotechnology, Santa Cruz, CA, USA) in 0.5% goat serum PBS. After rinsing three times for 5 min in 0.1% Tween-20-PBS followed by one rinse with PBS, the slides were incubated for 30 min with biotinylated secondary antibodies (Vectastatin ABC secondary antibody kit, PK6101, Vector Labs) in 5% goat serum PBS, rinsed and incubated for an additional 30 min at RT with ABC solution and subsequently developed in diaminobenzidine (DAB) solution containing 2% DAB buffer, 4% DAB and 2% H_2_O_2_ (DAB staining kit, SK-4100, Vector Labs). Apoptotic cells were counted in 10 fields per section at 1000× magnification and are referred to as counts per high magnification field (cpf) throughout the text.

Splenocytes were isolated by passing the tissue through a wire mesh, followed by homogenization of the cell suspension and then centrifugation for 5 min at 4 °C. Red blood cells were eliminated by hypo-osmotic lysis using distilled water. Splenocyte cell death was determined by staining with Annexin V and propidium iodide (PI) (#556570, BD Pharmingen, San Jose, CA, USA) and analyzed by flow cytometry, as previously described [62].

### 4.5. Lung Histology and Immunohistochemistry

Tissues were fixed in 10% formalin, embedded in paraffin and cut into sections. After deparaffinization, sections were stained using H&E, following the manufacturers’ instructions (Thermo Scientific, Waltham, MA, USA). To determine the cell types undergoing apoptosis in the lung, lung sections were first stained using active caspase-3 antibodies (1:330 dilution, #9661 Cell Signaling Technology, Danvers, MA, USA), followed by co-labeling with antibodies specific for different cell types. Slides were developed using the Benchmark LT automated IHC system from Ventana Medical Systems (Tucson, AZ, USA) as previously described [63]. Antibodies that detect cell-specific markers, such as endothelial cells (anti-CD31 antibody, 1:20 dilution, #MCA1738B, AbD Serotec, Raleigh, NC, USA), epithelial cells (anti-cytokeratin antibody 8.1, 1:100 dilution, (Sigma, St. Louis, MO, USA)) or neutrophils (anti-7/4 antibody, 1:100 dilution, #MCA771G AbD Serotec) were used. Color development for cell markers was obtained using the peroxidase substrate DAB. Analyses of co-localization of active caspase-3 and cell type markers was performed using the Nuance EX system (CRi, Wobur, MA, USA) attached to an Olympus BX40 microscope (Olympus America, Center Valley, PA, USA).

### 4.6. Leukocyte Lung Infiltration

Identification of neutrophils in fixed lungs was performed using a naphthol AS-D chloroacetate esterase (CAE) activity assay kit (#91C01KT, Sigma) according to the manufacturer’s instructions. Neutrophils were also identified also by IHC using the anti-7/4 antibodies (AbD Serotec, Raleigh, NC, USA) as described above. Ten fields per animal at 1000× magnification were counted and cpf were quantified as above.

### 4.7. Caspase-3 Activity in Lung Tissues

Lungs were rinsed in cold PBS and immediately frozen in liquid nitrogen. Frozen lung tissue was pulverized and then homogenized in hypotonic lysis buffer (25 mM HEPES [4-(2-hydroxyethyl)-1-piperazineethanesulfonic acid] pH 7.5, 5 mM MgCl_2_, 1 mM EGTA (ethylene glycol tetraacetic acid), 0.1 mM PMSF (PhenylMethaneSulfonyl Fluoride) and protease inhibitors chymosin, pepstatin, antipain and leupeptin, each at 2 µg/mL); using a Polytron homogenizer set at medium speed for 30 s. Homogenates were centrifuged at 12,000× *g* for 10 min at 4 °C. Protein lysates were incubated in a cytobuffer containing 20 µM DEVD-AFC (tetrapeptide Ac-Asp-Glu-Val-Asp-7-amino-4-trifluoromethylcoumarin flurogenic caspase-3 substrate from MP Biomedicals, Santa Ana, CA, USA) to determine caspase-3 activity, as previously described [64]. Released AFC was measured using a Cytofluor 400 fluorimeter (Filters: excitation 400 nm, emission 508 nm; Perspective Co., Framingham, MA, USA).

### 4.8. Immunodetection of Cytokines and Quantitative Real-Time PCR

Mouse MIP-2 and KC were measured in BALF collected at different times using ELISA kits (DuoSet #DY453 for KC and DuoSet #452 for MIP-2, R&D Systems, Minneapolis, MN, USA) according to the manufacturer’s instructions. Samples were read in a Dynatech MRX plate reader at 450 nm (Dynatech Laboratories, Chantilly, VA, USA) and data analyzed as previously reported [32].

RNA isolation and qRT-PCR of inflammatory cytokines was assessed as previously described [32]. The following primers were used for: MIP-2: sense, 5′-CTCAAGGGCGGTCAAAAAGTT-3′; and anti-sense, 5′-TGTTCAGTATCTTTTGGATGATTTTCTG-3′; for KC: sense, 5′-GCACCATGGCTGGGATT-3′; and anti-sense, 5′-CCTGAGGGCAACACCTTCAA-3′. The fold change of the mRNA evaluation of the relative copy number (RCN) and expression ratios of selected genes were normalized to the expression of two housekeeping genes: GAPDH (primers: sense, 5′-ACTTTGGTATCGTGGAAGGACT-3′; and anti-sense, 5′-GTAGAGGCAGGGATGATGTTCT-3′) and CAP (cyclic AMP-accessory protein primers: sense, 5′-ATTCCCTGGATTGTGAAATAGTC-3′; and anti-sense, 5′-ATTAAAGTCACCGCCTTCTGTAG-3′) and calculated with the equation: RCN = E^−Δ*C*t^ × 100, where E = the efficiency of PCR and *C*_t_ = *C*_t_ *target* − *C*_t_ *reference* (average of two housekeeping genes). PCR efficiency was calculated by the equation: E = 10 ^(−1/slope)^.

### 4.9. Cardiac Function and Mitochondrial Complex I Activity

*In vivo* cardiac dimension and contractile function were evaluated by M-mode echocardiography using a GE-Vivid 7 Dimension ultrasound imaging system and intra-operative epicardial probe (Model il3L; 14 MHz, Healthcare, Milwaukee, WI, USA) following the American Society of Echocardiography leading-edge method, as previously reported [65]. Data represent the average of 9 cardiac cycles from at least 3 separate scans. Left ventricular (LV) performance was assessed 24 h after treatment in isoflurane-anesthetized mice. LV M-mode measurements were taken with a sweep rate of 200 mm/s. Left ventricular fractional shortening percentage (FS%), a surrogate of systolic function, was calculated using the following formula: FS% = ((LVED *–* LVES)/LVED] × 100, where LVED means LV end-diastolic internal diameter and LVES means LV end-systolic internal diameter.

Mitochondrial Complex I activity was determined in cardiac tissue lysates. Heart tissue (~50 mg) was homogenized in PBS using a Polytron Homogenizer, centrifuged at 800× *g* and red blood cells removed by hypotonic rinse in 500 µL dH_2_O followed by centrifugation at 800× *g*. The pellets were resuspended in 100 µL lysis buffer (sucrose 50 mM, Tris 20 mM, 0.02% SDS, KCl 40 mM, EGTA 2 mM), incubated 20 min at 4 °C and centrifuged at 800× *g* for 10 min. Protein lysates (100 μg) were resuspended in 200 μL assay buffer containing 20 mM potassium phosphate pH 7.6, 2 mM NaN_3_, 0.8% sodium cholate and 16 mM ubiquitin. The reaction was initiated by the addition of 1.5 mM NADH, and 5 µg/mL rotenone was used as a specific inhibitor of Complex I. Complex I activity was determined by assessing the change in NADH absorbance at 340 nm every 10 s for 6 min using the EnSpire multimode plate reader (PerkinElmer, Waltham, MA, USA). Enzymatic units were calculated as previously described [33].

### 4.10. Statistical Analysis

Kaplan–Meier survival curve analysis, Student’s *t*-tests and one-way ANOVA were performed using GraphPad Prism Version 4.03 for Windows (GraphPad Software, San Diego, CA, USA). All data are presented as the mean ± SEM.
